# Screening of retail milk in Ontario for the presence of influenza A viral RNA

**DOI:** 10.14745/ccdr.v51i04a06

**Published:** 2025-04-03

**Authors:** Juliette Blais-Savoie, Winfield Yim, Jonathon D Kotwa, Lily Yip, Robert Kozak, Allison McGeer, Samira Mubareka

**Affiliations:** 1 Sunnybrook Research Institute, Toronto, ON; 2 Department of Laboratory Medicine and Pathobiology, University of Toronto, Toronto, ON; 3 Department of Microbiology, Mount Sinai Hospital, Toronto, ON

**Keywords:** highly pathogenic avian influenza virus, milk, H5N1, H5Nx

## Abstract

**Background:**

In April of 2024, studies in the United States (US) identified the presence of influenza A(H5N1) viral RNA in 20%–40% of commercially available pasteurized milk in the US, suggesting that cattle infections were widespread across the country.

**Methods:**

As an initial assessment of the situation in Ontario, 117 samples of pasteurized cow’s milk purchased from retail outlets in Ontario in April and May of 2024 were tested for the presence of influenza A viral RNA.

**Results:**

No influenza A viral RNA was detected.

**Conclusion:**

The Canadian Food Inspection Agency has subsequently developed an ongoing surveillance system for testing commercially available pasteurized milk and raw milk at processing plants in Canada.

## Introduction

Bovine infections with the H5N1 highly pathogenic avian influenza (HPAI) virus were first identified in the United States (US) in March 2024, as part of an ongoing panzootic of clade 2.3.4.4b H5Nx, which has infected a range of mammalian species across six continents ((1)). In late April, multiple studies identified the presence of H5N1 HPAI viral RNA (vRNA) in commercially available pasteurized milk across the US, suggesting that infections in dairy cattle were much more widely disseminated than had been initially believed ((2)). Genomic analysis suggested that the cattle outbreaks originated from a single interspecies spillover from wild birds, followed by sustained cow-to-cow transmission for several months prior to initial identification ((3)).

In April, a human case of H5N1 HPAI was identified in a dairy worker who had presumed exposure to infected cattle, indicating a new risk factor for infection in humans ((4)).

### Issue identification

Ontario has the second-largest number of dairy farms and dairy cattle in Canada ((5)) and borders several US states with active outbreaks, including Michigan. Cross-border movement of cattle occurs on a regular basis. As commercial milk testing in the US strongly suggested many more cattle outbreaks than had been detected, and genomic analysis suggested that they had been occurring for several months, it was not clear whether cryptic infections and milk contamination might also be occurring in Canada.

## Methods

A pilot study to assess the presence of influenza A vRNA in commercially available pasteurized milk products in the province of Ontario was undertaken. This study included pasteurized cow’s milk purchased from commercial vendors. Cream products (5% fat content or higher), flavoured milk products (e.g., chocolate milk), cheese and other dairy were excluded. Ultra-high-temperature and microfiltered milk were included. Samples were sourced from microbiology laboratory staff and other related research staff in Ontario, transported in clean or sterile containers, then aliquoted and frozen at −80°C pending testing.

Samples were thawed and vortexed, then diluted 1:4 (vol/vol) in sterile phosphate buffered saline (PBS) solution ((6)). Ribonucleic acid (RNA) extraction was conducted using NucliSENS® easyMAG® following generic protocol 2.0.1 with 140 μL input volume and 40 μL elution volume. Armored RNA enterovirus (arm-ent) (Asuragen) spiking was used as an internal extraction control. Samples were tested by quantitative polymerase chain reaction (qPCR) on the QuantStudio 3 platform with the Luna® Universal One-Step RT-qPCR Kit (NEB #E3005). Samples were tested in duplicate with 5 μL of RNA template and the following cycling conditions with fast qPCR conditions: a hold stage of 55°C for 10 minutes and 95°C for 1 minute, followed by a PCR stage with 44 amplification cycles of 95°C for 10 seconds and 60°C for 30 seconds. The PCR assay multiplex included the US Centers for Disease Control and Prevention’s universal influenza A matrix gene primer and probe set ((7)) (below) and arm-ent primer and probe set.

1) CDC_FluA (probe): 5’ d FAM-TGCAGTCCTCGCTCACTGGGCACG-BHQ-1 3’

2) CDC_FluA_Forward: 5’ d GACCRATCCTGTCACCTCTGAC 3’

3) CDC_FluA_Reverse: 5’ d AGGGCATTYTGGACAAAKCGTCTA 3’

## Results

Limit of detection of the assay for vRNA in milk samples was determined by spiking 2% milk with 10-fold dilutions of influenza A H3N2 virus from 10^5^ plaque forming units (pfu)/mL to 10^1^ pfu/mL. Samples were then pasteurized at 72°C for no less than 16 seconds, as this is most common practice in Ontario ((8)) and has been shown to be effective for the inactivation of H5N1 influenza A virus. One study showed complete inactivation in seven of eight samples and a 4.44 log_10_ dilution in the eighth sample with this pasteurization method ((9)). The limit of detection for the assay with the above protocol is a viral concentration of 10^2^ pfu/mL prior to pasteurization. Optimization experiments showed no significant difference in influenza A vRNA recovery between whole milk and 2% milk after dilution in PBS. The following processes were tested during assay optimization and provided no significant improvements to limit of detection: centrifuging milk samples prior to extraction at 4°C and 6,000 times gravity (xg) for five minutes, filtering milk samples prior to extraction with 0.45 μm filter, increasing extraction input volume to 600 μL or 200 μL, adding additional wash steps to the extraction (easyMAG specific B 2.0.1 protocol), conducting a magnetic bead clean-up on RNA template prior to PCR and decreasing RNA template volume to 3 μL, 2.5 μL, 2 μL or 1 μL.

**Figure 1:**
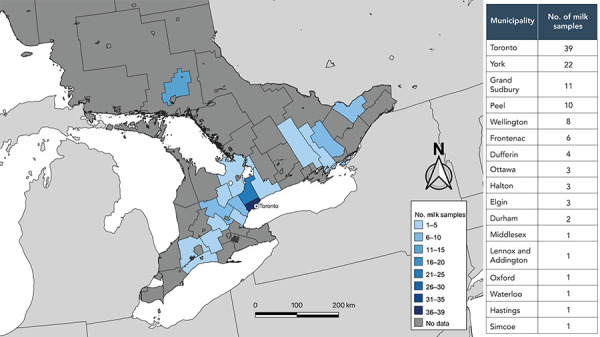
Locations of purchase of commercially available milk samples^a^ ^a^ This map of the province of Ontario, Canada, indicates the upper and single tier municipalities where the commercial milk samples for this study were purchased. Cities of Toronto, Ottawa and Greater Sudbury are single tier; other municipalities are upper tier, meaning they include more than one city/town. Colour indicates the number of samples purchased at each municipality. The city of Toronto is labeled

The Sunnybrook Research Institute received 117 commercially available pasteurized cow’s milk samples, collected from April 24, 2024, to May 6, 2024, from 17 different municipalities across Ontario (**Figure 1**). Overall, 28 brands were sampled with the following breakdown in types of products: six skim milk, 24 1% milk, 56 2% milk and 31 whole (3%–3.25%) milk, with best before dates ranging from April 15, 2024, to August 28, 2024. Two samples were provided as controls from milk that had been frozen at −20°C for approximately 18 months. All samples tested negative for influenza A virus target by qPCR with internal extraction control (arm-ent) target cycle threshold values ranging from 31–40.

## Discussion

### Limitations

Influenza A vRNA was not detected in any of the commercially available pasteurized Ontario milk products included in this study. Sampling in this study had limitations. Milk from all regions of the province was unable to be included and the absence of publicly available data on milk processing and distribution in Ontario meant that milk could not be collected from a representative sample of farms; while almost all milk sold in Ontario is from farms and processing plants in Ontario, the possibility that some milk may have come from Québec could not be excluded. The sample size of this pilot study did not rule out the possibility of H5N1 HPAI circulating in Ontario cattle. However, if the sample used in the study could be replicated, the 95% upper confidence limits suggest that fewer than 4% of milk samples would be contaminated. The difference between this upper limit of 4% and the 20% and 40% identified in US commercial pasteurized milk samples by Speakman *et al.* ((2)) and Tarbuck *et al*. (6), respectively, suggested significantly lower contamination rates in Ontario compared to the US.

Subsequent to this study, as of October 2024, multiple additional US states have identified outbreaks in cattle for a total of 299 cases in cattle across 14 states ((1)). In addition, a total of seven human cases of H5N1 HPAI in dairy farm workers have been reported in four states (Texas, Michigan, Colorado and California) ((4,10,11)). Additionally, a single human case of H5N1 HPAI has been reported in Missouri in a person with no known exposure to cattle, wild animals or birds ((11)).

## Conclusion

Several studies have also identified that the pasteurization of milk, while it may not result in the complete killing of A(H5N1) HPAI, is likely to successfully mitigate the risk of infection to consumers ((8)).

In Canada, no cases of H5N1 HPAI in either cattle or humans have been identified as of October 10, 2024. While the current absence of evidence of H5N1 in Canadian cattle is reassuring, continued monitoring is imperative. Ontario is continuing to subtype all human influenza cases requiring hospital admission and surveillance for influenza-associated infection in cattle continues. The Canadian Food Inspection Agency, in collaboration with the Public Health Agency of Canada, has established ongoing surveillance of both commercial pasteurized milk and raw bulk milk from commercial processing plants. As of October 1, 2024, 1,211 samples of commercial pasteurized milk (307 from Ontario) and 272 samples of raw bulk milk (68 from Ontario) have been tested and found to be negative ((12)).

This pilot demonstrated that a One Health collaborative approach, with input from human and veterinary microbiologists and epidemiologists, as well as public health experts, could provide rapid preliminary data to support a situational assessment of a zoonotic risk. In this particular situation, government laboratories were able to establish ongoing surveillance and later able to access raw bulk milk for sampling, which is likely to be more sensitive and will be accompanied by metadata accessible to the responsible agencies regarding the specific sources of milk. Thus, further work by this group is not needed. However, rapid collaborative responses may be a resource for Canadians in future public health investigations.
